# Highly efficient and precise base editing in discarded human tripronuclear embryos

**DOI:** 10.1007/s13238-017-0458-7

**Published:** 2017-08-19

**Authors:** Guanglei Li, Yajing Liu, Yanting Zeng, Jianan Li, Lijie Wang, Guang Yang, Dunjin Chen, Xiaoyun Shang, Jia Chen, Xingxu Huang, Jianqiao Liu

**Affiliations:** 1 Department of Reproductive Medicine, Third Affiliated Hospital of Guangzhou Medical University, Guangzhou, 510150 China; 2 School of Life Science and Technology, ShanghaiTech University, Shanghai, 201210 China; 3 Institute of Immunology, PLA, Third Military Medical University, Chongqing, 400038 China; 4 Key Laboratory for Reproduction and Genetics of Guangdong Higher Education Institutes, Key Laboratory for Major Obstetric Diseases of Guangdong Province, Third Affiliated Hospital of Guangzhou Medical University, Guangzhou, 510150 China

**Dear Editor,**

CRISPR/Cas9 is a powerful tool for genome editing (Komor et al., 2017). Recently, it has been employed in several attempts to edit the human embryos (Liang et al., 2015; Kang et al., 2016; Tang et al., 2017). A major technical concern particularly relevant in studies involving human embryos is the potential off-target effects (Callaway, 2016; Plaza Reyes and Lanner, 2017). Consequently, development of safer genome editing strategy in human embryos is highly anticipated (Cyranoski and Reardon, 2015). The off-target mutation result in part from Cas9-mediated double strand break (DSB) of DNA. Recently, base editing (BE) without the introduction of DSB has been achieved. The key design for BE is to use a catalytically inactive Cas9 to recruit the cytidine deaminase APOBEC to target sequences, leading to conversion of C to T within a window of approximately five nucleotides (Komor et al., 2016). Therefore, BE is apparently determined by additional features of the target sequence and offers a potentially safer approach for genome editing. Here we report the initial technical assessment of applying BE3, base editor 3 (Komor et al., 2016), in discarded human tripronuclear embryos.

We targeted two human gene sites, HEK293 site 4 and RNF2 (Komor et al., 2016). BE3 and sgRNAs were prepared *in vitro* as described (Shen et al., 2014), and microinjected into the cytoplasm of the tripronuclear zygotes with the concentration of one hundred nanogram BE3 and fifty nanogram sgRNA per microliter. The zygotes were collected 48 h after microinjection, with the embryos containing different numbers of cells ranging from 1 to 8 (Table S1). In total, 8 zygotes for each of the two targets (#1–8 for HEK293 site 4, #9–16 for RNF2) were collected (Fig. 1A). Whole genome of each individual sample was amplified and used as the template for further analysis.

**Figure 1:**
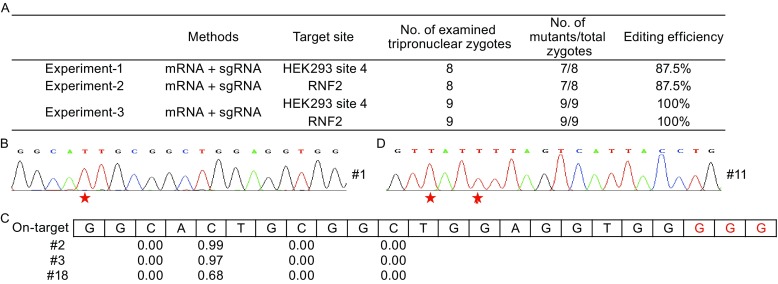
**Highly efficient base editing by BE3 in discarded human tripronuclear embryos**. (A) Summary of the information of the experiments. (B) The representative sequence chromatogram of site4 from sample #1. The sequence was the targeted sites. The red star indicated the conversion of C to T. (C) Summary of the deep sequencing of the on-target site for HEK293 site4. The editing efficiency of every C within the target site was indicated. The PAM was highlighted in red. (D) The representative sequence chromatogram of RNF2 from sample #11. The sequence was the targeted sites. The red star indicated the conversion of C to T

To detect the efficiency of base editing, the region around the target sites was amplified and analyzed initially by the T7EN1 cleavage assay. For HEK293 site 4, we did not detect any cleavage bands in any of the samples (Fig. S1A). However, sequencing of the bulk PCR products revealed C to T conversion at the sixteenth base distal from the PAM in 7 of the samples (#1–6, #8) (Figs. 1B and S1B), which is in accordance with the original report in human cell lines (Komor et al., 2016). We cloned 3 (#1–3) of the 8 bulk PCR products and sequenced multiple colonies from each primary product. For PCR products #2 and #3, each clone sequenced displayed C to T substitution, while PCR product #1 yielded one wildtype genotype besides the identical mutation genotypes (Fig. S1C), indicating highly efficient editing.

To more carefully analyze the on-target editing effects, deep sequencing was applied to samples #2 and #3. In total, more than 3 M clean reads for each sample were generated. The results showed that only the 16th nucleotide distal from the PAM completely carried C to T conversion with the efficiency as high as 0.97 for sample #3, and 0.99 for sample #2. No other nucleotide alteration was detected (Fig. 1C). Besides, no on-target indel was found (Table S2). These results demonstrated the BE led to highly precise and efficient genome editing in human embryos.

The same tests were performed for RNF2. T7EN1 cleavage bands were detected in 7 out of the 8 samples (#9–13, #15–16) (Fig. S2A). Sanger sequencing of PCR products confirmed C to T conversion in the 7 samples with cleavage (Figs. 1D and S2B). To further analyze the editing, 3 samples (#10–12) were selected for genotyping by TA cloning and subsequent sequencing. As reported before (Komor et al., 2016), in most cases, 2 cytosines (at the 18th and 15th nucleotide distal from the PAM) were simultaneously mutated to T, and triple C to T conversion (at the 18th, 15th, and 9th nucleotide distal from the PAM) also occurred (sample #10 and #12) (Fig. S2C). Collectively, these results demonstrated highly efficient and precise on-target base editing by BE3 in human embryos.

We next tried to mutate the two genes simultaneously in the tripronuclear zygotes. To avoid possible toxicity, the concentration of each sgRNA was lowered to 25 nanogram per microliter. Nine embryos (#17–25) were collected and the target sites were analyzed by sequencing (Fig. 1A). For HEK293 site 4, the expected substitution in the sixteenth base distal from the PAM was observed in all samples (Fig. S3A), although the wild type genotype was also detectable in a few samples (Fig. S3B). A sample (#18) was randomly selected for on-target analysis by deep sequencing. The results showed that the conversion rate in the 16th C was about 0.68, which was consistent with the results of bulk PCR products sequencing (Fig. 1C). For RNF2, mutations were detected in all samples although with lower efficiency than the HEK293 site 4 site in some samples (Fig. S4). Similar to previous results in human cell lines (Komor et al., 2016), besides C→T conversion, we observed rare C→G substitution (sample #21) and C→A conversion (sample #22). This result was verified by re-sequencing of the cloned products. Similar to the situations of targeting RNF2 alone shown above, triple substitution was found in sub-clones from sample #23. These results further demonstrated efficient base editing of BE3 in human embryos.

The off-target mutagenesis is a major concern for all genome editing approaches (Tsai and Joung, 2016). Since no off-target for the sgRNA against RNF2 was detected by the GUIDE-seq (Tsai et al., 2015), we therefore focused on the off-target analysis when editing HEK293 site 4. We selected 7 off-target sites according to the GUIDE-seq results, and amplified the regions around the selected off-target sites for samples #2, #3, #5, #8, #18, #20, #22, and #25. All PCR products were sequenced and compared with the reference sequence. The results showed, among the 56 sites of the 8 samples, two-site substitution for off-target site #3 and one-site substitution for off-target site #4 were found on sample #3 (Fig. 2A and 2B). No off-target mutagenesis was detected in other samples (Fig. S5).

**Figure 2:**
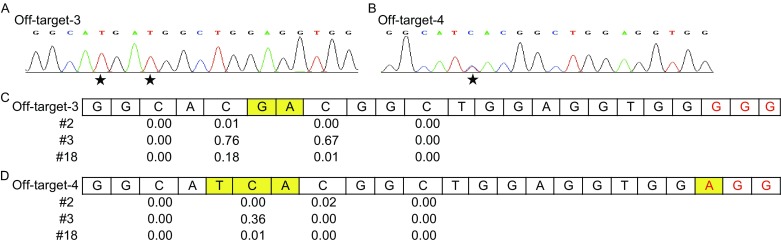
**Low off-target mutation detection by BE3 in discarded human tripronuclear embryos**. (A) The representative sequence chromatogram of off-target site #3 detected in sample #3. The black stars indicated the conversion of C to T. (B) The representative sequence chromatogram of off-target site #4 detected in sample #3. The black stars indicated the conversion of C to T. (C) Summary of the deep sequencing of the off-target site #3. The editing efficiency of every C within the target site was indicated. The PAM was highlighted in red. The different nucleotides compared with the target site were highlighted in yellow. The PAM was highlighted in red. (D) Summary of the deep sequencing of the off-target site #4. The editing efficiency of every C within the target site was indicated. The PAM was highlighted in red. The different nucleotides compared with the target site were highlighted in yellow. The PAM was highlighted in red

To further characterize the possible off-target by BE in human embryos, deep sequencing analysis, which detects targeting effect with the most sensitivity, was performed on 5 reported off-target sites (#1, #3, #4, #6, #8) (Komor et al., 2016). The sample #3 with off-target mutagenesis, together with randomly selected sample #2 and #18 were subjected for analysis. As described above, more than 3 M clean reads for each off-target site of every sample were generated. The C→T conversion in these off-target sites were firstly calculated. As expected, off-target site 3 containing two-site alteration and site 4 containing one-site alteration were demonstrated in sample #3 (Fig. 2C and 2D), and further confirmed by sequencing the PCR products. In addition, a C→T conversion at the 16th nucleotide distal from the PAM of the off-target site 3 was detected in sample #18 with the frequency of 0.18.

Considering the possible indel mediated by BE at off-target sites, then, the indel frequency was calculated for all the off-target sites. The results showed, the indels were found in off-target site 3 of all the 3 analyzed samples with the frequency of 0.09 for sample #2, 0.10 for sample #3, and 0.05 for sample #18, respectively (Table S2). We also detected indels in some other off-target sites with the frequency of 0.01 at off-target site 4 for #2 and #3, 0.02 at off-target site 6 for #2, and off-target site 8 for #3, respectively (Table S2), which might be derived from the noise sequence. Taken together, the above results showed that BE3 induces near perfect gene editing in the target site with extremely low off-target mutagenesis for human embryos. Nevertheless, more research on other aspects such as toxicity associated with the use of BE3 in human embryos will be warranted in the future.

This study represents the first successful application of base editor in human embryos. This exciting strategy is highly efficient and safer than editing based on DSBs. Therefore, our line of research may have future implications considering that nearly one thousand human genetic diseases involve T→C or A→G mutations.

## FOOTNOTES

We thank members of Huang and Liu labs for helpful discussions. We are grateful to Mr. Ming Zhao from Shanghai Institute of Hematology & State Key Laboratory of Medical Genomics, Rui Jin Hospital, Shanghai Jiao Tong University School of Medicine for his excellent technical assistance, and Dr. Jianghuai Liu from Nanjing University for excellent language editing. This works is supported by the Innovation of Science and Technology Commission of Guangzhou, China (201604020075) and National Natural Science Foundation of China (Grant No. 31471400).

The authors declare no competing financial interests.

## Electronic supplementary material

Below is the link to the electronic supplementary material.

Supplementary material 1 (PDF 1945 kb)
