# Supplementary material for: Highly efficient and precise base editing in discarded human tripronuclear embryos
Source: Protein Cell. 2017 Aug 19;8(10):776–9. doi: 10.1007/s13238-017-0458-7 (PMC5636751; doi:10.1007/s13238-017-0458-7)
Supplement: Supplementary file 1 — Supplementary material 1 (PDF 1945 kb) [file 13238_2017_458_MOESM1_ESM.pdf]

Supplementary Information

Materials and Methods

Discarded human tripronuclear zygotes

All of the used tripronuclear zygotes were donated by the patients undergoing IVF treatment at the Center for Reproductive Medicine, the Third Affiliated Hospital of Guangzhou Medical University, who have signed the informed consent before the sample collection. The study was approved by the Ethics Committee of the Third Affiliated Hospital of Guangzhou Medical University.

Plasmid construction

BE3 plasmid was reported by David R. Liu [1]. The sgRNA expression plasmid for i*n vitro* transcription was constructed as reported protocol [2]. Briefly, the oligos targeting the HEK293 site4 and RNF2 were synthesized, annealed, and inserted into the linearized pUC57-T7sgRNA (Addgene 51132).

***In vitro* transcription of BE3 and sgRNAs**

In vitro transcription of BE3 and sgRNAs were performed as described previously with some modifications [3]. In brief, BE3 plasmid was extracted using plasmid midi kits (TIANGEN, DP107-02), and linearized by digestion with Bbs I (NEB, R0539S). The linearized plasmid was purified with PCR purification kit (Axygen, AP-PCR-500G) and *in vitro* transcribed using mMESSAGE mMACHINE T7 Ultra Kit (Ambion, Life Technologies, AM1345). The template for sgRNA transcription was amplified from the constructed Puc57-T7 sgRNA plasmid with the primers: F: 5'-TCTCGCGCGTTTCGGTGATGACGG-3'; R: 5'-AAAAAAAGCACCGACTCGGTGCCACTTTTTC-3'. The purified PCR product (564 bp) was used as the template to transcript *in vitro* using the MEGAshortscript Kit (Ambion, Life Technologies, AM1354). The sgRNA was purified with the MEGAclear Kit (Ambion, Life Technologies, AM1908).

Embryo injection and culture

After 16-18 hours of IVF, the tripronuclear zygotes were picked under microscope. The concentration of BE3 mRNA was diluted into 100 ng/μL, and the sgRNA was diluted into 50 ng/μL. Microinjection was performed using an inverted microscope equipped with a microinjector and micromanipulators. With 2-day culture after injection, embryos were transferred individually into 20 μL droplets of the acidic Tyrode's solution (Solarbio, Beijing, China) using a denudation

pipet (Vitrolife, Gottenburg, Sweden) at 25 ± 1 ℃ and monitored continuously using an inverted microscope. The digestion time was strictly controlled to avoid complete removal of zona pellucida (ZP). After partial ZP digestion, the embryos were taken out and washed immediately with 100 μL of G-MOPS-Plus medium (Vitrolife, Gottenburg, Sweden) to remove the digestion solution.

Whole genome amplification of single embryo

Whole genomes of all the digested individual embryo were amplified using Discover-sc Single Cell Kit (Vazyme, N601-01). The amplified genomes were diluted with 50 times volume water for next PCR reaction.

T7EN1 cleavage assay

The sequence around the target sites was amplified and purified, and the used primers were listed in Table S2. Because base editing could result in the same alteration in the tripronuclear zygotes, wild type PCR products were added into the PCR products of the injected embryos with the same quantity. A total 200 ng of the mixed PCR products were annealed and digested with T7EN1, then separated by a 2.5% agarose gel.

Off-target assay

Seven sites with detected off-target mutagenesis by GUIDE-seq in HEK293 site4 were selected [1]. The primers used to amplify the off-target sequence were listed in Table S2. The PCR products of the experiment-1 and experiment-3 samples were sequenced directly.

Deep sequencing of on-target and off-target site

Shorter sequence (200-350 bp) containing the target sites or off-target sites were amplified with high-fidelity polymerase. All of the used primers were listed in Table S3. The PCR products were purified from the agarose gel to remove non-specificity sequence. The PCR products were submitted to Novogene Bioinformatics Institute (http://www.novogene.com/en/) for deep sequencing using Hiseq2500-PE250. Each sites had about 3 M clean reads.

REFERENCES

1 Komor AC, Kim YB, Packer MS, Zuris JA, Liu DR. *Nature* 2016; **533**:420-424.

2 Shen B, Zhang W, Zhang J *et al. Nature methods* 2014; **11**:399-402.

3 Shen B, Zhang J, Wu H *et al. Cell research* 2013; **23**:720-723.

Supplementary Figures

Figure S1 Detection of BE-mediated base editing of HEK293 site4 in samples from Experiment 1.

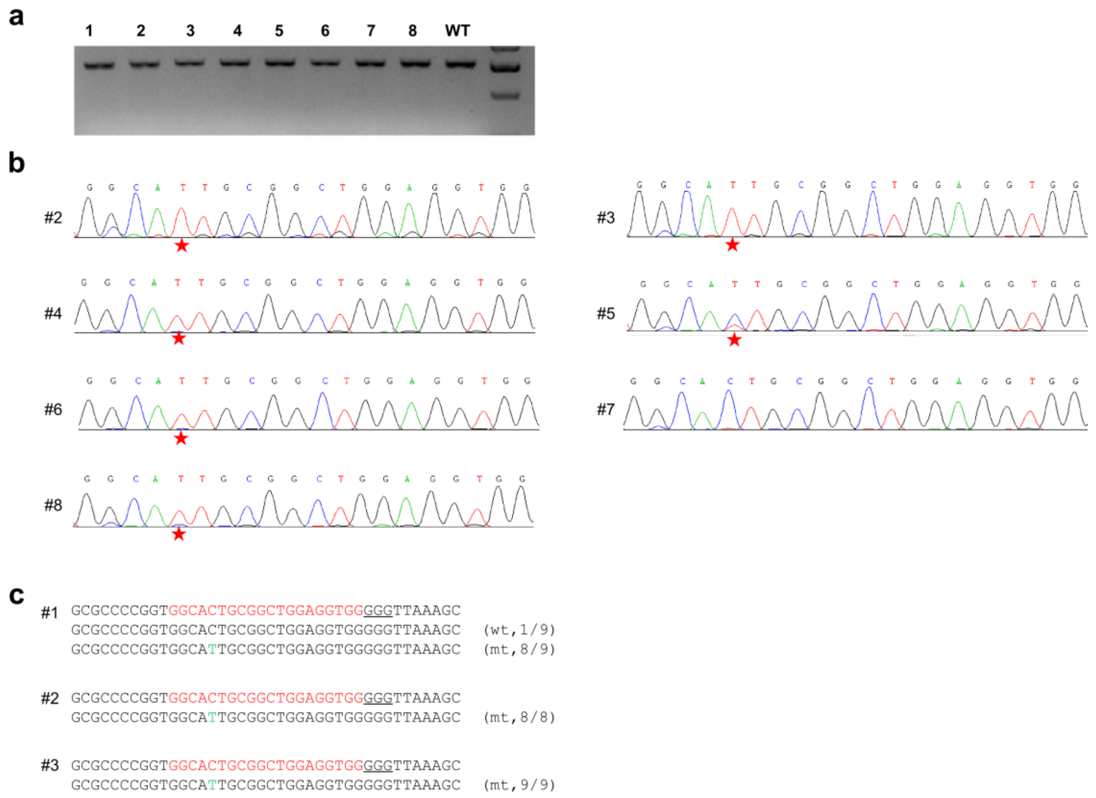

(a) Detection of BE-mediated base editing of HEK293 site4 by T7EN1 assay.

(b) The representative sequence chromatogram of HEK293 site4 in samples from Experiment 1. The sequence was the targeted site. The red stars indicated the conversion of C to T.

(c) Sequences of modified HEK293 site4 locus. TA clones of the PCR products were analyzed by Sanger sequencing. The PAM sequence is underlined. The targeting sequences are highlighted in red; the substituted nucleotides in green. N/N represents positive colonies out of total sequenced.

Figure S2 Detection of BE-mediated base editing of RNF2 in samples from Experiment 2.

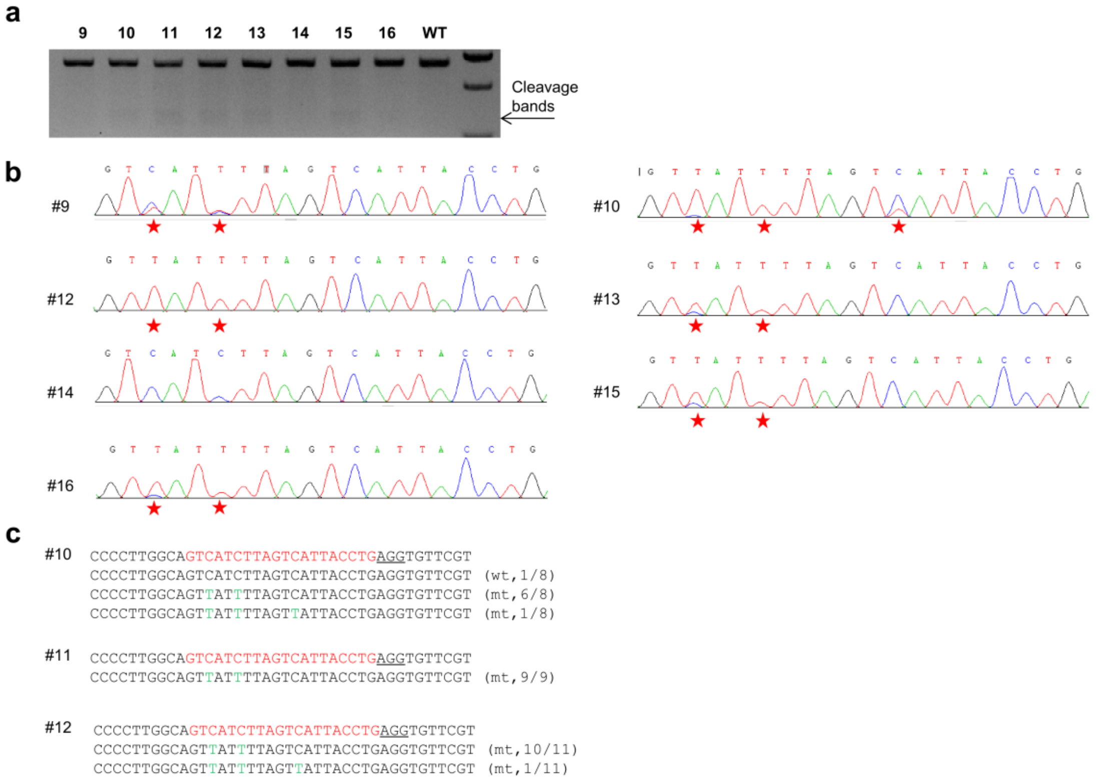

(a) Detection of BE-mediated base editing of RNF2 by T7EN1 assay.

(b) The representative sequence chromatogram of RNF2 in samples from experiment 2. The sequence was the targeted site. The red stars indicated the conversion of C to T.

(c) Sequences of modified RNF2 locus. TA clones of the PCR products were analyzed by Sanger sequencing. The PAM sequence is underlined. The targeting sequences are highlighted in red; the substituted nucleotides in green. N/N represents positive colonies out of total sequenced.

Figure S3 Detection of BE-mediated base editing of HEK293 site4 in samples from Experiment 3.

a

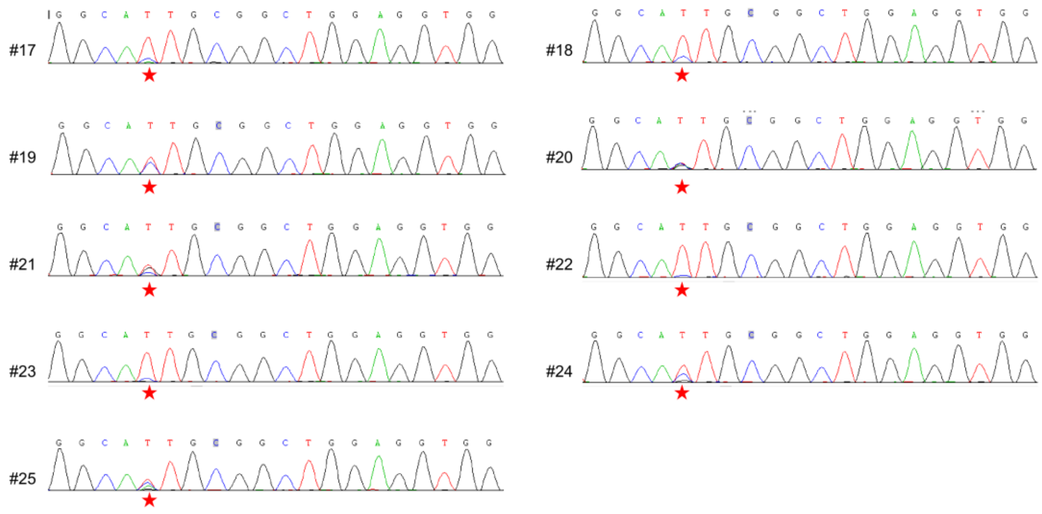

b

```
#18 GCGCCCCGGTGGCACTGCGGCTGGAGGTGGGGGTTAAAGC
    GCGCCCCGGTGGCACTGCGGCTGGAGGTGGGGGTTAAAGC  (wt,3/14)
    GCGCCCCGGTGGCATTGCGGCTGGAGGTGGGGGTTAAAGC  (mt,10/14)
    GCGCCCCGGTGGTATTGCGGCTGGAGGTGGGGGTTAAAGC  (mt,1/14)

#20 GCGCCCCGGTGGCACTGCGGCTGGAGGTGGGGGTTAAAGC
    GCGCCCCGGTGGCACTGCGGCTGGAGGTGGGGGTTAAAGC  (wt,2/10)
    GCGCCCCGGTGGCAGTGCGGCTGGAGGTGGGGGTTAAAGC  (mt,4/10)
    GCGCCCCGGTGGCATTGCGGCTGGAGGTGGGGGTTAAAGC  (mt,4/10)

#22 GCGCCCCGGTGGCACTGCGGCTGGAGGTGGGGGTTAAAGC
    GCGCCCCGGTGGCATTGCGGCTGGAGGTGGGGGTTAAAGC  (mt,5/5)

#23 GCGCCCCGGTGGCACTGCGGCTGGAGGTGGGGGTTAAAGC
    GCGCCCCGGTGGCACTGCGGCTGGAGGTGGGGGTTAAAGC  (wt,1/6)
    GCGCCCCGGTGGCATTGCGGCTGGAGGTGGGGGTTAAAGC  (mt,5/6)

#25 GCGCCCCGGTGGCACTGCGGCTGGAGGTGGGGGTTAAAGC
    GCGCCCCGGTGGCACTGCGGCTGGAGGTGGGGGTTAAAGC  (wt,1/9)
    GCGCCCCGGTGGCAATGCGGCTGGAGGTGGGGGTTAAAGC  (mt,6/9)
    GCGCCCCGGTGGCATTGCGGCTGGAGGTGGGGGTTAAAGC  (mt,2/9)
```

(a) The representative sequence chromatogram of HEK293 site4 in samples from Experiment 3. The sequence was the targeted site. The red stars indicated the conversion of C to T.

(b) Sequences of modified of HEK293 site4 locus. TA clones of the PCR products were analyzed by Sanger sequencing. The PAM sequence is underlined. The targeting sequence is highlighted in red; the substituted nucleotide in green. N/N represents positive colonies out of total sequenced.

Figure S4 Detection of BE-mediated base editing of RNF2 in samples from Experiment 3.

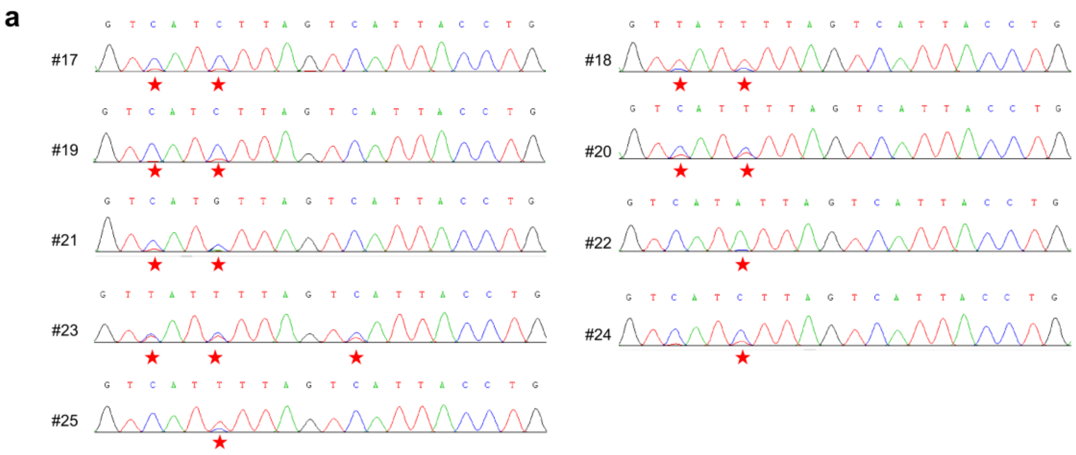

b

```
#18  CCCCTTGGCAGTCATCTTAGTCATTACCTGAGGTGTTCGT
     CCCCTTGGCAGTCATCTTAGTCATTACCTGAGGTGTTCGT (mt.2/14)
     CCCCTTGGCAGTTATTTTAGTCATTACCTGAGGTGTTCGT (mt,11/14)
     CCCCTTGGCAGTCATTTTAGTCATTACCTGAGGTGTTCGT (mt,1/14)

#20  CCCCTTGGCAGTCATCTTAGTCATTACCTGAGGTGTTCGT
     CCCCTTGGCAGTCATCTTAGTCATTACCTGAGGTGTTCGT (wt.9/13)
     CCCCTTGGCAGTTATTTTAGTCATTACCTGAGGTGTTCGT (mt,3/13)
     CCCCTTGGCAGTCATGTTAGTCATTACCTGAGGTGTTCGT (mt,1/13)

#22  CCCCTTGGCAGTCATCTTAGTCATTACCTGAGGTGTTCGT
     CCCCTTGGCAGTCATCTTAGTCATTACCTGAGGTGTTCGT (wt,2/8)
     CCCCTTGGCAGTCATATTAGTCATTACCTGAGGTGTTCGT (mt,6/8)

#23  CCCCTTGGCAGTCATCTTAGTCATTACCTGAGGTGTTCGT
     CCCCTTGGCAGTCATCTTAGTCATTACCTGAGGTGTTCGT (wt,1/8)
     CCCCTTGGCAGTCATCTTAGTTATTACCTGAGGTGTTCGT (mt,1/8)
     CCCCTTGGCAGTTATTTTAGTCATTACCTGAGGTGTTCGT (mt.2/8)
     CCCCTTGGCAGTTATTTTAGTTATTACCTGAGGTGTTCGT (mt,4/8)

#25  CCCCTTGGCAGTCATCTTAGTCATTACCTGAGGTGTTCGT
     CCCCTTGGCAGTCATCTTAGTCATTACCTGAGGTGTTCGT (wt.9/13)
     CCCCTTGGCAGTTATTTTAGTCATTACCTGAGGTGTTCGT (mt,1/13)
     CCCCTTGGCAGTCATTTTAGTCATTACCTGAGGTGTTCGT (mt,1/13)
     CCCCTTGGCAGTCATGTTAGTCATTACCTGAGGTGTTCGT (mt,2/13)
```

(a) The representative sequence chromatogram of RNF2 in samples from Experiment 3. The sequence was the targeted sites. The red stars indicated the conversion of C to T.

(b) Sequences of modified RNF2 locus. TA clones of the PCR products were analyzed by Sanger sequencing. The PAM sequence is underlined. The targeting sequences are highlighted in red; the substituted nucleotides in green. N/N represents positive colonies out of total sequenced.

Figure S5 The representative sequence chromatogram of the potential off-target sites of HEK293 site4.

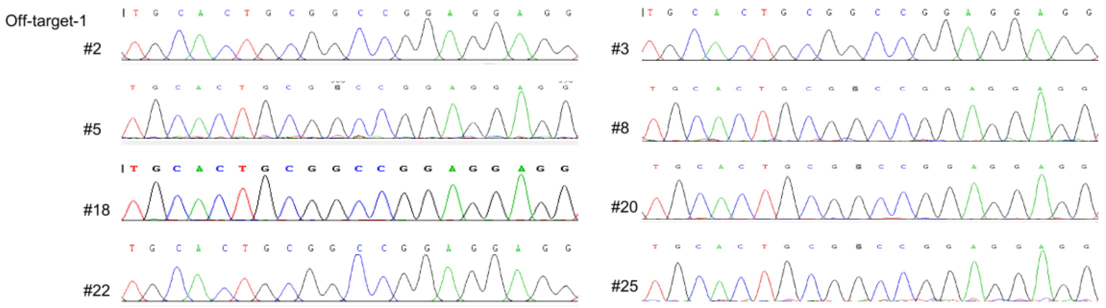

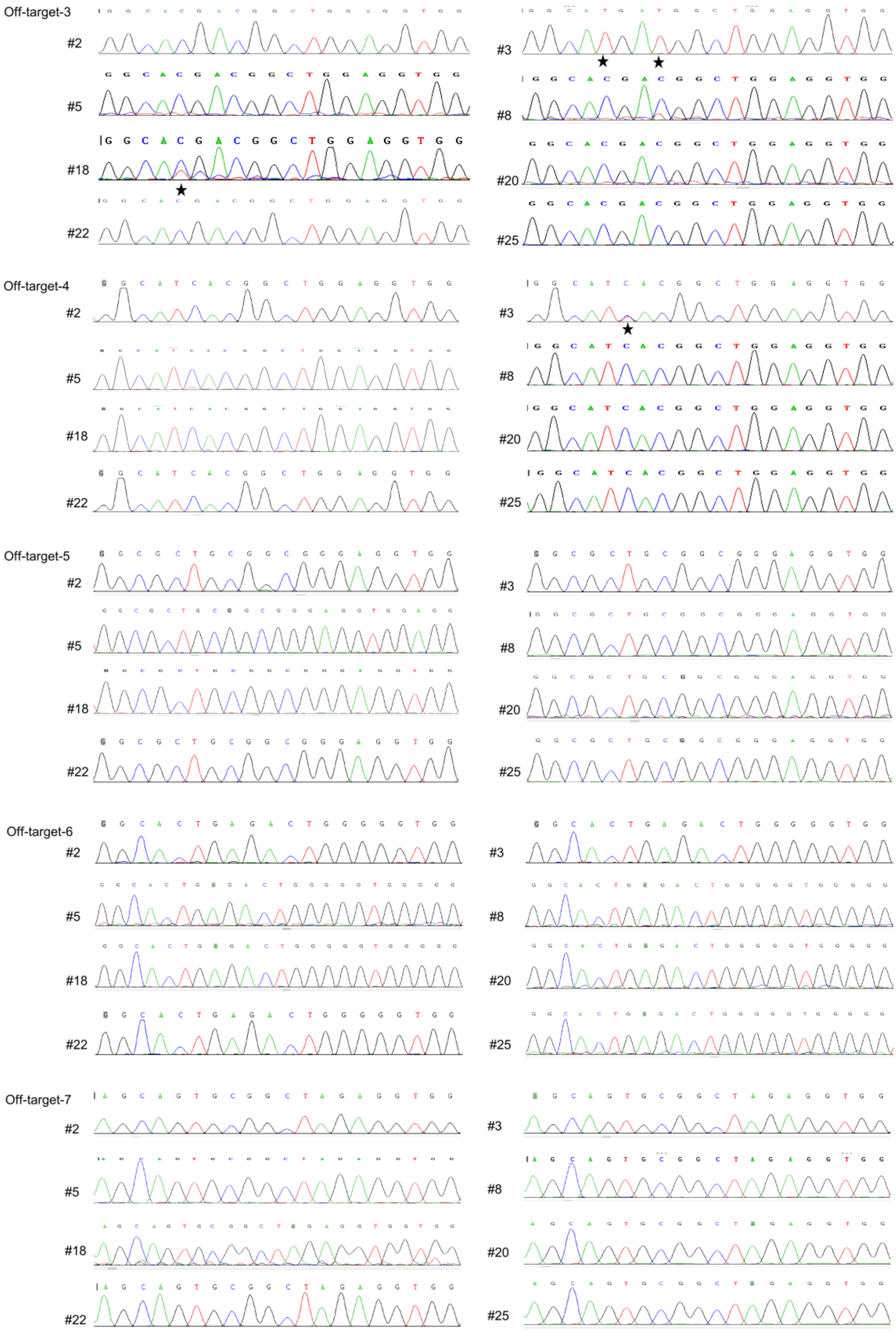
Off-target-3
#2
#3
#5
#8
#18
#20
#22
#25
Off-target-4
#2
#3
#5
#8
#18
#20
#22
#25
Off-target-5
#2
#3
#5
#8
#18
#20
#22
#25
Off-target-6
#2
#3
#5
#8
#18
#20
#22
#25
Off-target-7
#2
#3
#5
#8
#18
#20
#22
#25

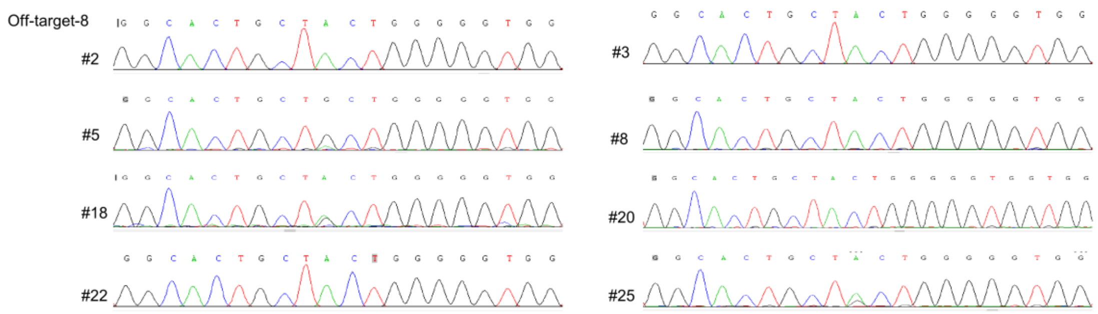

Seven potential off-target sites mostly homologous to HEK293 site4 were amplified from embryo #2, #3, #5, #8, #18, #20, #22 and #25. The sequence was the targeted sites. Black stars indicated the conversion of C to T in the off-target site.

Figure S6 Summary of the deep sequencing.

Off-target-1	T	G	C	A	C	T	G	C	G	G	C	C	G	G	A	G	G	A	G	G	T	G	G
#2			0.00		0.00			0.00			0.00	0.00											
#3			0.00		0.00			0.00			0.00	0.00											
#18			0.00		0.00			0.00			0.00	0.00											

Off-target-6	G	G	C	A	C	T	G	A	G	A	C	T	G	G	G	G	G	T	G	G	G	G	G
#2			0.00		0.00						0.00												
#3			0.00		0.00						0.00												
#18			0.00		0.00						0.00												

Off-target-8	G	G	C	A	C	T	G	C	T	A	C	T	G	G	G	G	G	T	G	G	T	G	G
#2			0.00		0.00			0.00			0.00												
#3			0.00		0.00			0.00			0.00												
#18			0.00		0.00			0.00			0.00												

Summary of the deep sequencing of the off-target site #1, #6, and #8. The editing efficiency of every C within the target site was indicated. The PAM was highlighted in red. The different nucleotides compared with the target site in yellow.

Table S1 Summary of the microinjection

Experiment number	Serial number	Cell number after injection	The concentration of BE3 and sgRNA
Experiment-1	1	3	BE3: 104.2ng/μL<br>HEK293 site4-sgRNA:50.9ng/μL
	2	4	
	3	2	
	4	3	
	5	2	
	6	4	
	7	3	
	8	2	
Experiment-2	9	1	BE3: 104.2ng/μL<br>RNF2-sgRNA:48.5ng/μL
	10	3	
	11	8	
	12	6	
	13	3	
	14	1	
	15	1	
	16	4	
Experiment-3	17	9	BE3: 104.2ng/μL<br>HEK293 site4-sgRNA:25.5ng/μL<br>RNF2-sgRNA:25ng/μL
	18	8	
	19	5	
	20	7	
	21	9	
	22	4	
	23	7	
	24	1	
	25	1	

Table S2 Summary of the Indels at on-target and off-target sites

	On-target	Off-target-1	Off-target-3	Off-target-4	Off-target-6	Off-target-8
#2	0.00	0.00	0.09	0.01	0.02	0.00
#3	0.00	0.00	0.10	0.01	0.00	0.02
#18	0.00	0.00	0.05	0.00	0.00	0.00

Table S3 Sequences of the primers

The name of the primers	Sequence (5'-3')	Usage
BE3-RNF2-T7-F	TAGGGTCATCTTAGTCATTACCTG	sgRNA plasmid construction
BE3-RNF2-T7-R	AAACCAGGTAATGACTAAGATGAC	
BE3-SITE4-T7-F	TAGGGGCACTGCGGCTGGAGGTGG	
BE3-SITE4-T7-R	AAACCCACCTCCAGCCGCAGTGCC	
BE3-RNF2-F	AGGAGGACTTGCCCAACTTT	genotyping
BE3-RNF2-R	CTGTGTCAGAACATGCTGGAA	
BE3-SITE4-F	TGAGCATCTACTGAGATCCT	
BE3-SITE4-R	GCCGCAGCCACTCAGAT	
SITE4-off-1-F	TGCTGAAGCCGGATTGTGAG	Off target detection by T7EN1
SITE4-off-1-R	GCTGGCCATTCCGGATGATT	
SITE4-off-3-F	CACACAGGAAAACCACGCTG	
SITE4-off-3-R	TCAGAAGCCTGAAAGAGGCG	
SITE4-off-4-F	CAGGTGTTCAGCTTTGCCAC	
SITE4-off-4-R	CATGTAACAGGGGTGGGCAT	
SITE4-off-6-F	CCTGGGTTCTTAAACACTAGGCT	
SITE4-off-6-R	GGCCTGGGTCTGTTATGGTC	
SITE4-off-8-F	CTCTTGGTACACCTCAGCCG	
SITE4-off-8-R	GCAGAAACTGCTCCCTCTGT	
SITE4-deepseq-F	GGGCGGCTTCTCCCTCAGTCA	Deep sequencing
SITE4-deepseq-R	CCTTTCAACCCGAACGGAGACACA	
SITE4-deepoff1-F	TGAGACTCATAGCTGGGGCT	
SITE4-deepoff1-R	TAAAAGCAGCTCTGGCCTCC	
SITE4-deepoff3-F	GTCTGAGGCTCGAATCCTGG	
SITE4-deepoff3-R	CCTTGGCATTGTCCCAGCTA	
SITE4-deepoff4-F	TGGGATGGAATCACCTGCAC	
SITE4-deepoff4-R	GGGGAGTAGAGACAGGCTCA	
SITE4-deepoff6-F	TCCGCCTCCCCTAATCTTG	
SITE4-deepoff6-R	GAGTGTGGAATCTGTACCCTGTG	
SITE4-deepoff8-F	GTACACCTCAGCCGTGTGTC	
SITE4-deepoff8-R	GCCCTGACTGGACCCTATTC	
